# Estimation of Crop Growth Parameters Using UAV-Based Hyperspectral Remote Sensing Data

**DOI:** 10.3390/s20051296

**Published:** 2020-02-27

**Authors:** Huilin Tao, Haikuan Feng, Liangji Xu, Mengke Miao, Huiling Long, Jibo Yue, Zhenhai Li, Guijun Yang, Xiaodong Yang, Lingling Fan

**Affiliations:** 1 Key Laboratory of Quantitative Remote Sensing in Agriculture, Ministry of Agriculture and Rural Affairs, P. R. China, Beijing Research Center for Information Technology in Agriculture, Beijing 100097, China; 15755515505@163.com (H.T.); mengkemiao17@163.com (M.M.); longhl@nercita.org.cn (H.L.); yuejibo@smail.nju.edu.cn (J.Y.); lizh@nercita.org.cn (Z.L.); guijunyang1@163.com (G.Y.); yangxd@nercita.org.cn (X.Y.); p17301156@stu.ahu.edu.cn (L.F.); 2 School of Geodesy and Geomatics, Anhui University of Science and Technology, Huainan 232001, China; ljxu@aust.edu.cn; 3 National Engineering Research Center for Information Technology in Agriculture, Beijing 100097, China; 4 Beijing Engineering Research Center for Agriculture Internet of Things, Beijing 100097, China

**Keywords:** vegetation index, red-edge parameters, above-ground biomass, leaf area index, stepwise regression, partial least squares regression

## Abstract

Above-ground biomass (AGB) and the leaf area index (LAI) are important indicators for the assessment of crop growth, and are therefore important for agricultural management. Although improvements have been made in the monitoring of crop growth parameters using ground- and satellite-based sensors, the application of these technologies is limited by imaging difficulties, complex data processing, and low spatial resolution. Therefore, this study evaluated the use of hyperspectral indices, red-edge parameters, and their combination to estimate and map the distributions of AGB and LAI for various growth stages of winter wheat. A hyperspectral sensor mounted on an unmanned aerial vehicle was used to obtain vegetation indices and red-edge parameters, and stepwise regression (SWR) and partial least squares regression (PLSR) methods were used to accurately estimate the AGB and LAI based on these vegetation indices, red-edge parameters, and their combination. The results show that: (i) most of the studied vegetation indices and red-edge parameters are significantly highly correlated with AGB and LAI; (ii) overall, the correlations between vegetation indices and AGB and LAI, respectively, are stronger than those between red-edge parameters and AGB and LAI, respectively; (iii) Compared with the estimations using only vegetation indices or red-edge parameters, the estimation of AGB and LAI using a combination of vegetation indices and red-edge parameters is more accurate; and (iv) The estimations of AGB and LAI obtained using the PLSR method are superior to those obtained using the SWR method. Therefore, combining vegetation indices with red-edge parameters and using the PLSR method can improve the estimation of AGB and LAI.

## 1. Introduction

Crop growth can also reflect soil conditions, nutritional status [1]. Above-ground biomass (AGB) and the leaf area index (LAI) are two of the main crop growth parameters, and play important roles in monitoring crop growth in agricultural management [2,3]. The timely and accurate estimation of crop growth parameters can provide a strong basis for the formulation of timely agricultural policies and food trade, and can also allow the estimation of the loss of crop yield caused by meteorological disasters and pests. Additionally, the accurate estimation of growth parameters can improve the accuracy of growth monitoring but also of growth modeling [4,5]. However, the traditional method of measuring crop growth parameters is destructive to crops [6], requires large amounts of manpower, material resources, and time, and has a small application range.

Remote sensing technology has attracted attention as a way to better estimate crop growth parameters due to its ability to provide timely, dynamic, macro-scale observations. This method involves estimating crop growth parameters using spectral data of the crop canopy [7,8], and has been widely applied [9,10,11,12]. Remote sensing technology is divided into ground-based, low-altitude, and high-altitude. For ground-based remote sensing, non-imaging ground-feature spectrometers are usually used. Due to the limited height of the platform, ground-feature spectrometers and cannot easily be used to generate orthophotos. Meanwhile, satellites can acquire image data over large areas, providing data with very high spatial resolution; however, they have long operating cycles, are affected by changes in weather and cloud cover, and high maintenance and operation costs. Low-altitude unmanned aerial vehicle (UAV) platforms are simple to operate, and have a higher spatial resolution than satellites [13,14,15,16,17,18,19,20]. Additionally, the flight requirements of UAVs are simpler than those of aircraft. UAV-based remote sensing technology has been applied in farmland management. The spectral parameters obtained from UAV-based cameras can be used to estimate crop growth parameters. At present, the sensors which are carried by UAVs are mostly Red-Green-Blue cameras and multispectral cameras, and, due to the small number of sensor bands in such cameras, the acquired spectral information is limited [21,22]. However, using hyperspectral sensors, which have more bands, more spectral data related to crop growth parameters can be obtained, thereby allowing a better estimation of growth parameters [23,24,25,26,27,28,29,30].

Vegetation indices (VIs) are obtained using a combination of two or more spectral bands. Usually, the spectral bands are combined mathematically. VIs can simply and effectively reflect the status of vegetation, and are widely used to estimate vegetation structure and physiological characteristics such as ground biomass, the LAI, and chlorophyll content. [6,9,10,31,32]. Based on crop characteristics and environmental impacts, researchers have developed many VIs to estimate crop growth parameters. For example, the optimized soil-adjusted vegetation index (OSAVI) has been used to obtain accurate estimates of the LAI [32]. Furthermore, the linear combination index (LCI) has been shown to allow accurate estimates of AGB [6]. Accurate estimates of crop parameters have also been obtained using a combination of a vegetation index and different methods. For example, Koppe et al. [33] used multiple regression to estimate winter-wheat biomass using VIs and microwave coefficients as estimation model factors, and obtained accurate results. Moreover, Jin et al. [34] used a VI and radar parameter to accurately estimate the LAI and biomass of winter wheat using partial least squares regression (PLSR). Additionally, it is possible to monitor crop growth using hyperspectral data from UAVs [35].

Compared with RGB and multispectral cameras, hyperspectral cameras can obtain more waveband information and can therefore better reflect the physiological and biochemical information of crops. However, the sensitivity of the waveband information obtained by hyperspectral sensors is different from the sensitivity of the crop growth parameters. Therefore, the reflectance of vegetation varies in different waveband ranges. For example, due to the absorption of chlorophyll and scattering by leaves and the canopy, the spectral reflectance of vegetation increases rapidly from a low value to a high value in the range of 670–750 nm, which is called the “red edge” band. The reflectance behavior in the red-edge band is very different from that in other bands [36]. Additionally, since this band contains a large amount of crop information, it can be used to estimate crop growth parameters.

Many of the abovementioned studies used UAV-based remote sensing data to estimate crop growth parameters such as AGB and the LAI. However, these studies only used VIs or a combination of VIs and analytical methods to estimate crop growth parameters. Meanwhile, very few studies have conducted in-depth investigations of spectral bands information to explore the impact of spectral information on the estimation of growth parameters. Therefore, the purpose of this study was to evaluate the estimation of the AGB and LAI of winter wheat during multiple growth stages using VIs, red-edge parameters, and their combination. Specifically, we (i) estimated the AGB and LAI using only VIs, only red-edge parameters, and a combination of VIs and red-edge parameters; (ii) compared the use of stepwise regression (SWR) and partial least squares regression (PLSR) to estimate the AGB and LAI; and (iii) combined VIs and red-edge parameters to estimate and map the distribution of AGB and LAI over multiple growth stages. The structure of this article is as follows: Section 1 introduces the research area and experimental design, ground data acquisition and processing, and acquisition and processing of UAV-based hyperspectral data. Selection of vegetation indices and red-edge parameters are also discussed; Section 2 introduces the results and analysis described above; and Section 3 and Section 4 present a discussion and conclusions, respectively.

## 2. Materials and Methods

### 2.1. Research Area and Experimental Design

**Figure 1:**
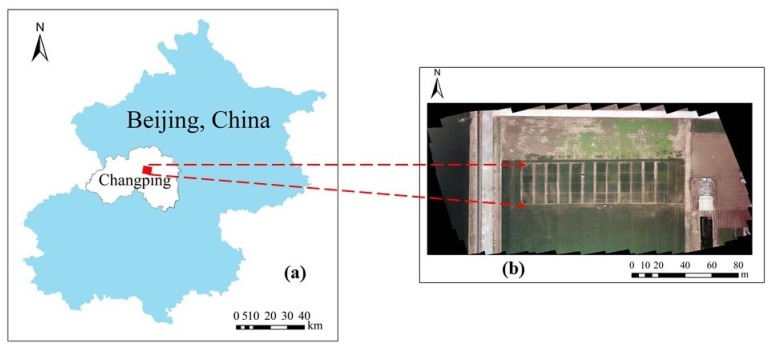
Location of the test area. (**a**) location of Changping District in Beijing; (**b**) image of test area acquired by unmanned aerial vehicle (UAV).

The research area is located in the National Precision Agriculture Research Demonstration Base in Xiaotangshan Town, Changping District, Beijing, China (40°10′48″–40°10′54″ N, 116°26′51″–116°26′53″ E, average altitude of about 36 m a.s.l.). The area has a warm temperate and semi-humid continental monsoon climate, with precipitation mostly occurring in summer and autumn, an average annual precipitation of about 42 mm, an average annual temperature of about 11.8 °C, and a large temperature difference between day and night. The previously planted crops were corn, and the soil type was fluvo-aquic, which was relatively fertile. The test area is shown in Figure 1.

**Figure 2:**
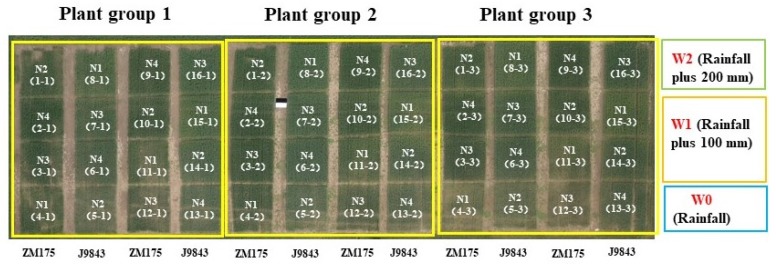
Experimental design. ZM175: winter-wheat variety Zhongmai 175; J9843: winter-wheat variety Jingmai 9843. N1, N2, N3, and N4 represent nitrogen treatments of 0, 195, 390, and 780 kg/ha, respectively.

Winter wheat was planted in 48 plots, each of which had an area of 48 m^2^. In order to increase the crop diversity of each experimental plot, two winter wheat varieties—Zhongmai 175 and Jingmai 9843—were selected; Zhongmai 175 was selected by the Chinese Academy of Agricultural Sciences, and Jingmai 9843 was selected by the Beijing Municipal Bureau of Agriculture. Different nitrogen and water treatments were used in each plot. Four nitrogen treatments were used—0 kg/ha (N1), 195 kg/ha (N2), 390 kg/ha (N3), and 780 kg/ha (N4)—and three water treatments were used—rainwater only (W0), rainwater plus normal irrigation (100 mm; W1), and rainwater plus twice normal irrigation (200 mm; W2). The planting density was 489 plants/m^2^. The specific test design is shown in Figure 2.

Remote sensing data were collected during four growth stages of winter wheat using a UAV carrying a hyperspectral sensor (see Section 2.3). Additionally, ground-based data acquisition was also carried out during each of these four stages (see Section 2.2). The ground-based and UAV-based data were acquired simultaneously on the same four days, corresponding to the winter-wheat jointing stage (21 April 2015), flag picking stage (26 April 2015), flowering stage (13 May 2015), and filling stage (22 May 2015).

### 2.2. Ground Data Acquisition and Processing

#### 2.2.1. Calculation of Leaf Area Index (LAI)

To measure the LAI, a total of 20 plants were harvested from each plot near the uniform growth area of the plot, and the total number of plants and the line spacing in the sampling area were measured. The samples were placed in a sealed paper bag and transported to a laboratory in the test base. In the laboratory, the stems and leaves were separated and all the leaves were placed on A4 paper. Then, the total area of each leaf was measured using a CI-203 Handheld Laser Leaf Area Meter (CID Bio-Science, Inc., Camas, WA, USA). Then, using this data, the LAI was obtained based on the counted number of single stems and the total number of stems per unit area in the sample plots. 

#### 2.2.2. Calculation of Above-Ground Biomass (AGB)

After calculating the LAI, the leaves and stems were placed in a paper bag, and the fresh biomass of the samples was measured using a high-precision balance (accuracy of 0.001 g). Then, the paper bag was placed in an oven at 80 ° C for 24 h to a constant mass state, and the sample was then weighed to determine the dry mass. Finally, the AGB was calculated by dividing the obtained dry mass by the surface area of the sample. 

### 2.3. Acquisition and Processing of UAV-Based Hyperspectral Data

Hyperspectral data were acquired on cloudless and windless days. The UAV platform used was a DJI S1000 UAV (SZ DJI Technology Co., Ltd., Sham Chun, Guangdong, China). The UAV has eight propellers, is equipped with two 18,000-mAh (25 V) batteries with a battery life of 30 min, and can maintain stability at low speed and low altitude; for the acquisition, the take-off mass was 6 kg, the flight altitude was 80 m, and the flight speed was 8 m/s.

The hyperspectral sensor carried by the UAV was a UHD 185-Firefly (Cubert GmbH, Ulm, Baden-Würtemberg, Germany), hereafter referred to as UHD185. The UHD185 is a full-frame non-scanning real-time imaging spectrometer with dimensions of 195 mm × 67 mm × 60 mm and a weight of 470 g. It acquires wavelengths from the visible to the near-infrared (450–950 nm) from 125 spectral bands, and can collect full-color images. The sampling interval of the hyperspectral data is 4 nm (Table 1). Atmospheric correction is generally required for remote sensing hyperspectral images; however, since the hyperspectral images obtained in this study were acquired under stable lighting conditions, atmospheric correction was not required. Following data acquisition, the Cubert Cube-Pilot software version 1.4 (Cubert GmbH) was used to perform two types of data (full-color images and hyperspectral cubes) fusion to form the hyperspectral images. The process of radiation correction involves converting the digital number (DN) value of the hyperspectral image to the surface reflectance [37]. Then, the Agisoft PhotoScan Professional version 1.1.6 software (Agisoft LLC, St. Petersburg, Russia) was used to stitch images [38].

### 2.4. Selection of Vegetation Indices and Red-Edge Parameters

#### 2.4.1. Selection of Vegetation Indices

The results of a large number of studies have shown that many vegetation indices are closely related to AGB and LAI. This study investigated the relationship between 20 VIs and AGB and LAI, the definitions of these VIs are shown in Table 2.

#### 2.4.2. Selection of Red-Edge Parameters

This study used five red-edge parameters: red edge position (REP), red edge amplitude (Dr), red edge area (SDr), minimum red-edge amplitude (Dr_min_), and the ratio of the red edge amplitude and the minimum red-edge amplitude (Dr/Dr_min_). The red-edge parameters were calculated according to the maximum first derivative [56,57,58]. REP is the wavelength of the maximum spectral reflectance curve slope, that is, the wavelength of the maximum first derivative of the spectrum in the range of 680–750 nm [56]; Dr is the first derivative of the red-edge position [57]; SDr is the sum of the first derivative of the spectrum of the red-edge region [58]; and Dr_min_ is the value of the minimum red-edge amplitude [57].

#### 2.4.3. Analysis Methods

SWR is a type of linear regression. When there are many independent variables, some of the factors may not have a large impact on the variables, and the independent variables may not be completely independent of each other. When introducing new variables, the previous model must be tested, insignificant variables must be eliminated, and this process must be repeated until new variables are not introduced. Additionally, the more independent variables the regression equation contains, and the smaller the error of the predicted value. If the equation has too many variables, the workload of forecasting will be greater, and if some of the predictors are not significantly correlated it will affect the accuracy of the prediction. Therefore, it is particularly important to choose the appropriate number of variables. PLSR is a combination of multiple linear regression, canonical correlation analysis, and principal component analysis. Models established using PLSR have advantages over traditional classical regression analysis, since in the PLSR method the best model is constructed by minimizing the sum of squares of errors [59]. In this study, PLSR was performed using the MATLAB 2018a software (MathWorks, Natick, MA, USA).

#### 2.4.4. Accuracy Evaluation

A total of 48 datasets (i.e., one for each plot) was obtained for each growth stage, with each dataset consisting of ground-measured and UAV-measured data. For the data analysis, 2/3 (32) of these datasets were used as the modeling set to build the model, and the remaining 1/3 (16) were used to validate the model. The accuracy of the AGB and LAI estimation models were evaluated using the coefficient of determination (*R^2^*), root-mean-square error (RMSE), and the normalized RMSE (NRMSE) [6]; a larger R^2^ indicates a better model fit, while a smaller RMSE and NRMSE indicate a higher model accuracy. The values of *R^2^*, RMSE, and NRMSE were calculated using Formulas (1), (2), and (3), respectively:

(1) $$R^{2}=1-\frac{\sum_{i=1}^{n}{\left(x_{i}-y_{i}\right)}^{2}}{\sum_{i=1}^{n}{\left(x_{i}-\bar{x}\right)}^{2}}$$

(2) $$RMSE=\sqrt{\frac{\sum_{i=1}^{n}{\left(y_{i}-x_{i}\right)}^{2}}{n}}$$

(3) $$NRMSE\left(\%\right)=\frac{RMSE}{\bar{X}}$$

where $x_{i}$ is the measured AGB or LAI for winter wheat, $\bar{x\;}$ is the average measured AGB or LAI, $y_{i}$ is the AGB or LAI predicted by the model, and *n* is the number of data points.

## 3. Results and Analysis

### 3.1. Correlation Analysis between Vegetation Indices and Red-Edge Parameters and AGB and the LAI

Correlation analysis was performed between the ground-measured AGB and LAI and (i) the 20 VIs and (ii) the five red-edge parameters, for different winter-wheat growth stages. The results are shown in Table 3. As shown in the table, for most of the VIs and red-edge parameters, the correlations with AGB and the LAI are significant at the *p* < 0.01 level. The correlation coefficients for both the VIs and the red-edge parameters are significantly different between the four growth stages; overall, the correlations between both AGB and the LAI and the VIs and red-edge parameters are stronger in the flowering stage than in the other three growth stages. The correlation between the vegetation indices and the red-edge parameters reached the highest, and the correlation at the filling stage began to be weak. Furthermore, the correlations between AGB and the VIs and red-edge parameters are significantly different to the correlations between the LAI and the VIs and red-edge parameters. For example, for the jointing stage, the correlation coefficients for AGB and the LAI are similar, with the most strongly correlated VI being the PBI and the most strongly correlated red-edge parameter being REP. For the flagging stage, the correlations between AGB and the VIs and red-edge parameters are stronger than those between the LAI and the VIs and red-edge parameters; for both AGB and LAI, the most strongly correlated VI is the LCI, and the most strongly correlated red-edge parameter is Dr/Dr_min_. For the flowering stage, stronger correlations are observed between AGB and the VIs and red-edge parameters than between the LAI and the VIs and red-edge parameters; for both AGB and the LAI, the most strongly correlated VI is the PBI and the most strongly correlated red-edge parameter is Dr. In general, for the filling stage, the correlations between the LAI and the VIs and red-edge parameters are stronger than those between AGB and the VIs and red-edge parameters; for AGB, the most strongly correlated VI and red-edge parameter is the LCI and Dr, respectively, while, for the LAI, the most strongly correlated VI and red-edge parameter is the PBI and Dr/Dr_min_, respectively. 

### 3.2. Relationship between Vegetation Indices and Red-Edge Parameters and AGB and LAI 

#### 3.2.1. Estimation of AGB and LAI Using Vegetation Indices 

A simple linear regression method was used to establish linear models between the studied VIs and the winter-wheat AGB and LAI, and the optimal vegetation index was selected for each growth stage based on the evaluation of the model performances (Table 4). For the modeling dataset, between the jointing stage and the flowering stage, the *R^2^* values of the models using the optimal VI increased from 0.47 to 0.70, and the NRMSE values decreased from 20.54 to 14.19%. Additionally, for the validation dataset, between the same growth stages, the R^2^ values of the models using the optimal VI increased from 0.60 to 0.72, and the NRMSE values decreased from 14.22 to 12.02%. That is, using the optimal VIs, the accuracy of the AGB estimates progressively improves between the jointing stage and the flowering stage. Meanwhile, between the flowering stage and the filling stage, the *R^2^* values of AGB estimates for the modeling dataset decreased from 0.70 to 0.45, while the *R^2^*values of AGB estimates for the validation dataset decreased from 0.72 to 0.65. Meanwhile, between the same two stages, the NRMSE of AGB estimates for the modeling dataset increased from 14.19 to 19.30%, while the NRMSE of AGB estimates for the validation dataset increased from 12.02 to 14.18%. For the LAI estimates, the *R^2^* values for the modeling dataset were similar between the jointing stage and the flagging stage, and the NRMSE values for the modeling dataset increased from 17.65 to 24.75% between the same two stages. From the flowering stage to the filling stage, for the modeling dataset, *R^2^* decreases from 0.65 to 0.63 and the NRMSE increases from 20.76 to 32.55%, that is, the model’s LAI estimation ability decreases.

#### 3.2.2. Estimation of AGB and LAI Using Red-Edge Parameters 

As described above for the VIs, linear regression analysis was used to determine the optimal red-edge parameters for the estimation of AGB and LAI for each growth stage. The *R^2^*, RMSE, and NRMSE values for the optimal red-edge parameters are shown in Table 5. For AGB, for the modeling dataset, the estimation progressively improves from the jointing stage to the flowering stage, with *R^2^* increasing from 0.49 to 0.62 and NRMSE decreasing from 22.57 to 16.04%, while, for the validation dataset, *R^2^* increases from 0.45 to 0.70 and NRMSE decreases from 16.87 to 12.46%. Meanwhile, for the modeling dataset, the estimation progressively worsens between the flowering stage and the filling stage, with *R^2^* decreasing from 0.62 to 0.45 and NRMSE increasing from 16.04 to 19.29%, while for the validation dataset *R^2^* decreased from 0.70 to 0.48 and NRMSE increased from 12.46 to 17.32%. From the jointing stage to the flagging stage, the LAI estimation ability decreases: for the modeling dataset, *R^2^*decreases from 0.57 to 0.51 and the NRMSE increases from 17.24 to 25.37%. Meanwhile, from the flagging stage to the filling stage, for the modeling dataset, the *R^2^* increases from 0.51 to 0.66 and the RMSE decreases from 1.16 to 0.53 kg/ha, the NRMSE increases from 25.37% to 31.09%. that is, the LAI estimation ability is enhanced. These results show that the estimation of AGB using red-edge parameters is superior to the estimation of LAI using these parameters.

#### 3.2.3. Estimation of AGB and LAI Using Combinations of Vegetation Indices and Red-Edge Parameters 

For each growth stage, a combination of the optimal VI and red-edge parameter for that growth stage (see Table 4 and Table 5) was used to estimate AGB and LAI. The results are shown in Table 6. As can be seen from a comparison between Table 4 and Table 5 and Table 6, the combinations of the optimal VI and red-edge parameter improved the estimation accuracy of AGB and LAI; the accuracy was improved by a small margin for the flagging and flowering stages, and was improved more significantly for the jointing and filling stages. For AGB, for the modeling dataset, the most accurate estimation was obtained in the flowering stage, the estimation accuracy increases progressively between the jointing stage and the flowering stage, and the estimation accuracy decreases between the flowering stage and the filling stage. For the estimation of LAI, for the modeling dataset, between the jointing stage and the flagging stage, *R^2^* decreased from 0.61 to 0.55 and the NRMSE increased from 16.42 to 24.33%; that is, the estimation worsened between these stages. Meanwhile, between the flagging stage and the filling stage, *R^2^*increased progressively from 0.55 to 0.69; however, the NRMSE also increased, from 24.33 to 29.94%, indicating a reduced estimation ability between these stages.

### 3.3. Estimating AGB and LAI Using Vegetation Indices and Red-Edge Parameters Combined with SWR and PLSR

**Figure 3:**
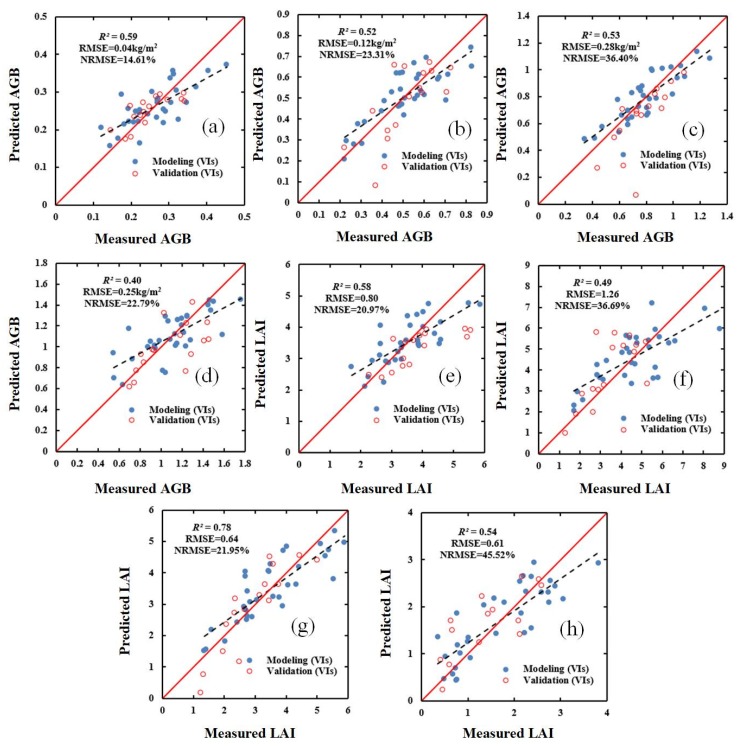
Relationship between ground-measured values of AGB (kg/m^2^) and LAI (m^2^/m^2^) in different growth stages of winter wheat and the values predicted based on vegetation indices (VIs) and stepwise regression (SWR): (**a**) AGB (jointing stage); (**b**) AGB (flagging stage); (**c**) AGB (flowering stage); (**d**) AGB (filling stage); (**e**) LAI (jointing stage); (**f**) LAI (flagging stage); (**g**) LAI (flowering stage); and (**h**) LAI (filling stage). *R^2^*: coefficient of determination; RMSE: root-mean-square error; NRMSE: normalized RMSE.

**Figure 4:**
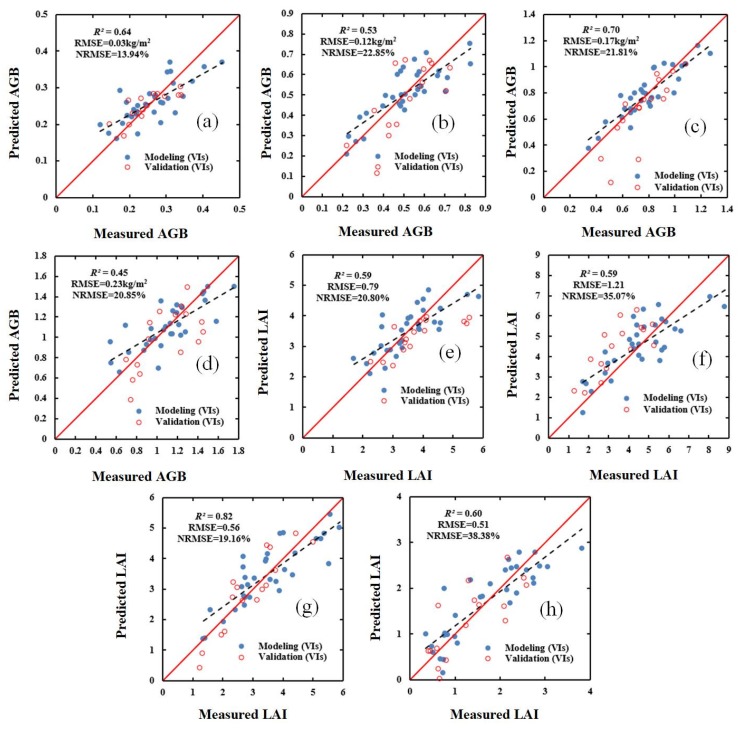
Relationship between ground-measured values of AGB (kg/m^2^) and LAI (m^2^/m^2^) in different growth stages of winter wheat and the values predicted based on VIs and PLSR: (**a**) AGB (jointing stage); (**b**) AGB (flagging stage); (**c**) AGB (flowering stage); (**d**) AGB (filling stage); (**e**) LAI (jointing stage); (**f**) LAI (flagging stage); (**g**) LAI (flowering stage); and (**h**) LAI (filling stage).

**Figure 5:**
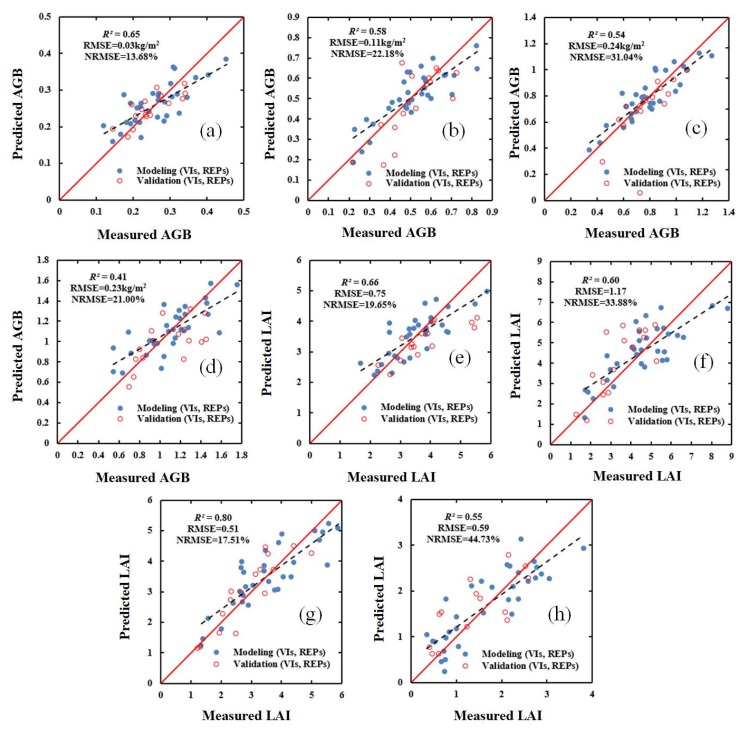
Relationship between ground-measured values of AGB (kg/m^2^) and LAI (m^2^/m^2^) in different growth stages of winter wheat and the values based on a combination of VIs and red-edge parameters (RPs) and SWR: (**a**) AGB (jointing stage); (**b**) AGB (flagging stage); (**c**) AGB (flowering stage); (**d**) AGB (filling stage); (**e**) LAI (jointing stage); (**f**) LAI (flagging stage); (**g**) LAI (flowering stage); (**h**) LAI (filling stage).

**Figure 6:**
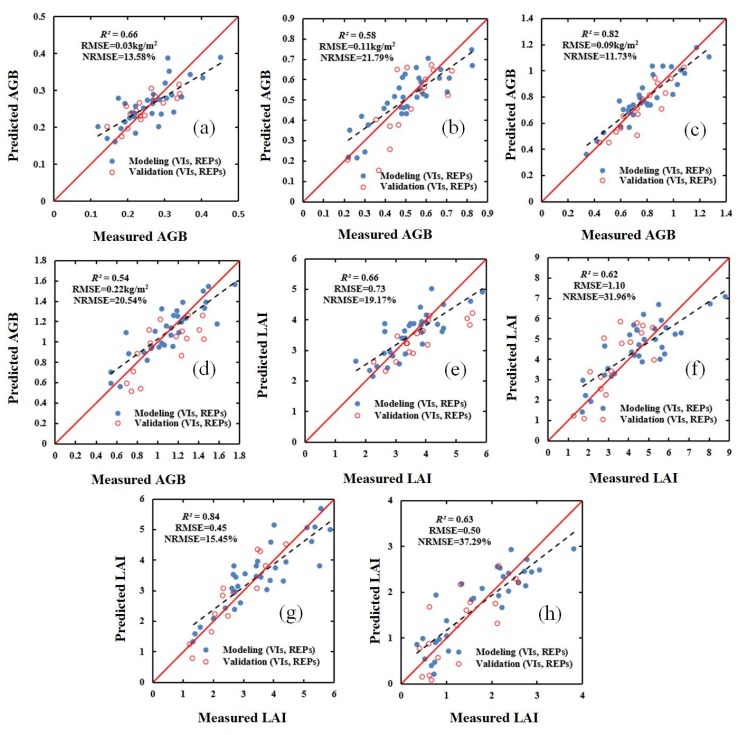
Relationship between ground-measured values of AGB (kg/m^2^) and LAI (m^2^/m^2^) in different growth stages of winter wheat and the values predicted based on a combination of VIs and RPs and PLSR: (**a**) AGB (jointing stage); (**b**) AGB (flagging stage); (**c**) AGB (flowering stage); (**d**) AGB (filling stage); (**e**) LAI (jointing stage); (**f**) LAI (flagging stage); (**g**) LAI (flowering stage); (**h**) LAI (filling stage).

Additionally, in order to assess the performances of SWR and PLSR for estimating crop parameters, the AGB and LAI were estimated for the different growth stages using (i) selected VIs, (ii) selected red-edge parameters, and (iii) a combination of selected VIs and red-edge parameters, using both the SWR and PLSR methods, respectively. As shown in Table 3, when building the model, the use of too many independent variables will lead to overfitting, and it is therefore necessary to ensure that an appropriate number of independent variables are used in the model, and that these variables have a high correlation. In order to achieve this, in this study, for each growth stage, we selected the seven vegetation indices and two red-edge parameters with the highest correlation coefficients. For the estimation of AGB, the following seven vegetation indices and two red-edge parameters were selected for each growth stage. For the jointing stage, the VIs PBI, LCI, PSSR, RARS, PSND, WDRVI, and MSR were chosen, and the red-edge parameters REP and Dr/Dr_min_ were chosen. For the flagging stage, the VIs LCI, PSRI, WDRVI, MSR, PBI, NDVI, and SR were chosen, and the red-edge parameters Dr/Dr_min_ and Dr were chosen. For the flowering stage, the VIs PBI, SR, PSSR, RARS, MSR, SPVI, and EVI2 were chosen, and the red-edge parameters Dr and SDr were chosen. Finally, for the filling stage, the VIs LCI, PBI, EVI2, PSSR, MSR, RDVI, and WDRVI were chosen, and the red-edge parameters Dr and SDr were chosen. Additionally, for the estimation of the LAI, the following seven vegetation indices and two red-edge parameters were also selected for each growth stage. For the jointing stage, the VIs PBI, LCI, PSSR, RARS, WDRVI, PSND, and MSR were chosen, and the red-edge parameters REP and Dr/Dr_min_ were chosen. For the flagging stage, the VIs LCI, PSSR, SR, RARS, MSR, PBI, and WDRVI were chosen, and the red-edge parameters Dr/Dr_min_ and Dr were chosen. For the flowering stage, the VIs PBI, PSSR, RARS, SR, MSR, LCI, and SPVI were chosen, and the red-edge parameters Dr and SDr were chosen. Finally, for the filling stage, the VIs PBI, LCI, WDRVI, MSR, SR, PSSR, and RARS were chosen, and the red-edge parameters Dr/Dr_min_ and Dr were chosen. The results are shown in Table 7 and Table 8 and Figure 3, Figure 4, Figure 5 and Figure 6. As shown in Table 7, for the modeling dataset and using the VIs, the best AGB estimation was obtained for the flowering stage for both the SWR and PLSR methods (SWR: *R^2^*= 0.74, RMSE = 0.11 kg/m^2^, NRMSE = 13.32%; PLSR: *R^2^*= 0.78, RMSE = 0.10 kg/m^2^, NRMSE = 12.26%); meanwhile, for the LAI, the estimation accuracy varies greatly between growth stages, with *R^2^* showing an upward trend between the jointing stage and the filling stage for both the SWR and PLSR methods; although the highest *R^2^* value is obtained for the filling stage when using the PLSR method, when considering all three accuracy metrics together, the best estimation of LAI is obtained during the flowering stage for both the SWR and PLSR methods.

Table 8 shows the estimates of AGB and LAI obtained using a combination of VIs and RPs using the SWR and PLSR methods, respectively. The results show that this estimation approach is superior to the approaches using either the VIs and RPs for both the SWR and PLSR methods. As shown in the table, the most accurate estimates were obtained for the flowering stage. For the SWR method, the largest *R^2^* value is 0.77 (AGB, flowering stage), and for the PLSR method the largest *R^2^* value is 0.80 (AGB, flowering stage). For the LAI estimates, the *R^2^* and NMRSE values tend to increase from the jointing stage to the filling stage, with the highest values of NMRSE being obtained for the filling stage for both the SWR and PLSR method and the highest values of *R^2^* being obtained for the flowering stage and filling stage for the SWR and PLSR methods, respectively.

In order to evaluate the effect of the models constructed using SWR and PLSR, the validation dataset was used to verify (i) the selected VIs, (ii) the selected red-edge parameters, and (iii) a combination of the selected VIs and the selected red-edge parameters. The results for the validation dataset are shown in Figure 3, Figure 4, Figure 5 and Figure 6. These results show that, compared with the estimation based on VIs only (Figure 3 and Figure 4), the estimation based on a combination of VIs and RPs is superior (Figure 5 and Figure 6).

The results show that using either the SWR or PLSR methods can significantly improve the accuracy of the estimation of AGB and LAI (Table 5 and Table 6). Additionally, in the four growth stages of winter wheat, the fit, accuracy, and model stability of the estimates of AGB and LAI obtained using the PLSR method are superior to those of the estimates obtained using the SWR method.

### 3.4. Construction of Spatial Distribution Map of AGB and LAI 

By comparing the AGB and LAI estimation models at different growth stages, it is found that the model using the PLSR method and a combination of VIs and RPs as model factors achieves the best estimation results. Therefore, this model was used to construct AGB and LAI distribution maps. 

**Figure 7:**
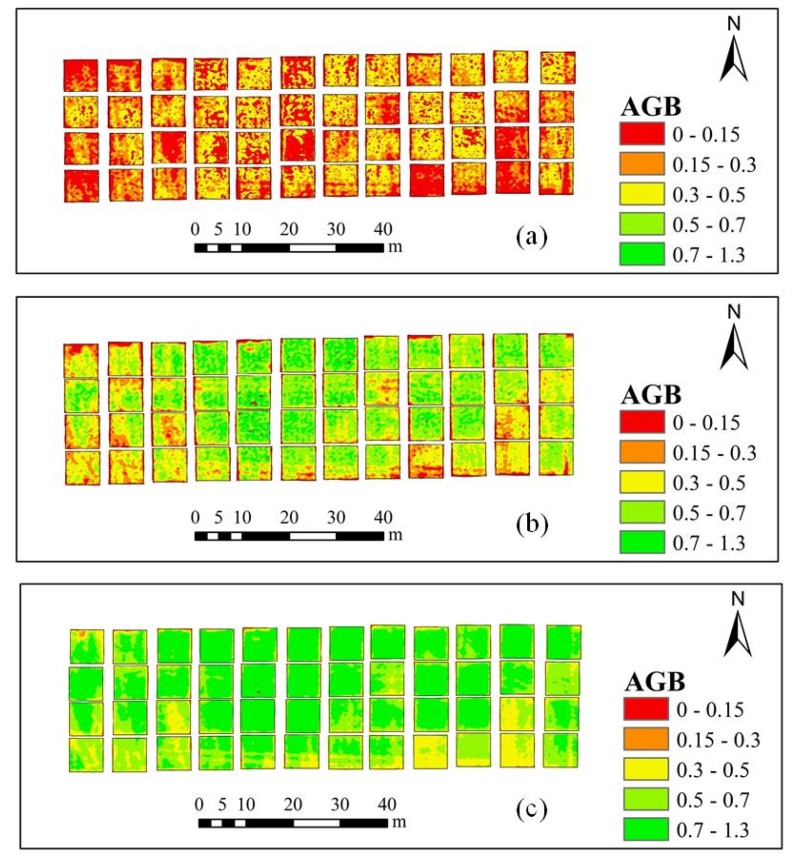
Maps showing the distribution of the estimated values of AGB (kg/m^2^) in the 48 study plots obtained using a combination of VIs and RPs and using the PLSR method. (**a**) jointing stage; (**b**) flagging stage; (**c**) flowering stage.

Maps showing the estimated spatial distribution of AGB in the winter-wheat jointing, flagging, and flowering stages is shown in Figure 7. As can be seen in the figure, from the jointing stage to the flowering stage, the estimated values of AGB gradually increase. In the jointing stage, the estimated AGB ranges from 0 to 0.5 kg/m^2^, with most values falling in the range 0 to 0.3 kg/m^2^. During the flagging stage, the estimated AGB ranges from 0 to 0.7 kg/m^2^. During the flowering stage, the estimated AGB ranges from 0 to 1.3 kg/m^2^, with most values falling in the range 0.5 to 1.3 kg/m^2^.

**Figure 8:**
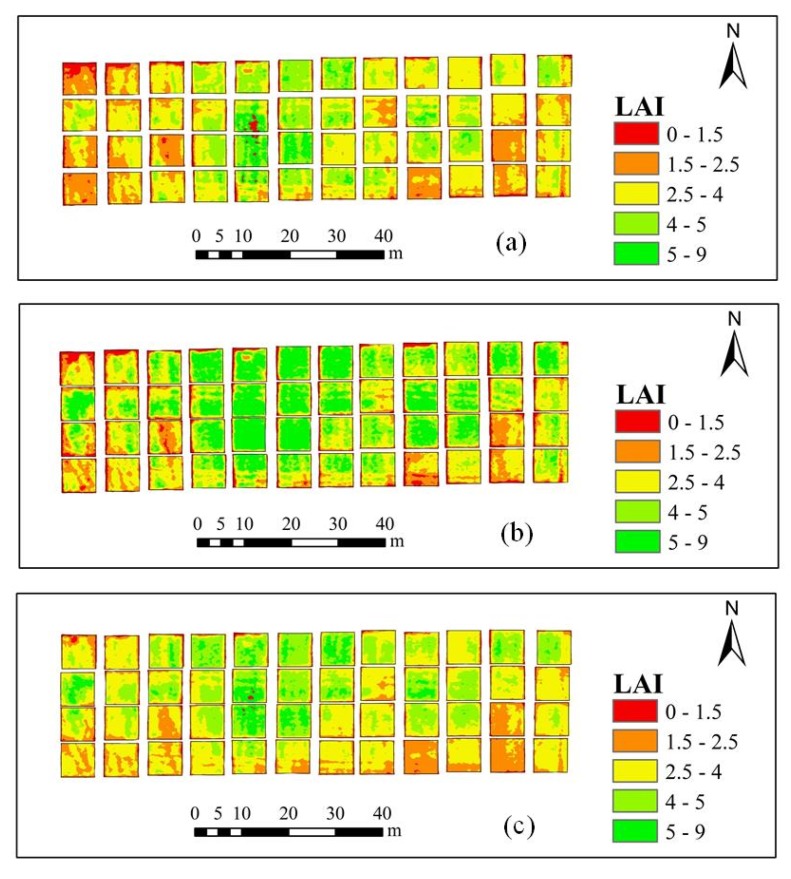
Maps showing the distribution of the estimated values of LAI (m^2^/m^2^) in the 48 study plots obtained using a combination of VIs and RPs and using the PLSR method. (**a**) jointing stage; (**b**) flagging stage; (**c**) flowering stage.

Similarly, the spatial distribution of LAI in the winter-wheat jointing, flagging, and flowering stages based on VIs and RPs and using the PLSR method is shown in Figure 8. As shown in the figure, the estimated LAI values increase between the jointing stage and the flagging stage, but decrease between the flagging stage and the flowering stage.

## 4. Discussion

### 4.1. Estimation of AGB and LAI Based on Vegetation Indices

In this work, the optimal VIs for the estimation of the AGB and LAI of winter wheat were determined for each growth stage. It was found that, from the jointing stage to the flowering stage, the optimal VIs for the estimation of AGB and LAI were the same for each growth stage, namely PBI for the jointing stage, LCI for the flagging stage, and PBI for the flowering stage. In the filling stage, the optimal VIs for the estimation of AGB and LAI were LCI and PBI, respectively. These findings show that the sensitivity of VIs to AGB and LAI is closely related to the crop growth stage. The PBI is constructed using wavelengths of 560 and 810 nm, while the LCI is constructed using wavelengths of 670, 710, and 850 nm. That is, both the PBI and LCI use visible and near-infrared wavelengths, and therefore contain the characteristics of vegetation at these wavelengths. Rama et al. [41] used the PBI to improve the inversion of the chlorophyll and nitrogen concentrations of crops. The present study proves that the PBI can also be used to obtain good estimates of crop AGB and LAI. Yue et al. [6] found that the LCI can be used to accurately invert AGB and LAI during the crop growth stage. Consistent with these findings, in the present study, the LCI was also found to have a strong estimation ability for AGB and LAI in different growing seasons, and was found to be the optimal vegetation index in multiple growing seasons. During growth, most photosynthetic products are stored in stems and leaves [60], which means that some VIs cannot be used to accurately estimate AGB. Therefore, determining the sensitivity of VIs to AGB and LAI in different growth stages is very important for the estimation of crop parameters based on UAV hyperspectral images.

### 4.2. Estimation of AGB and LAI Based on Red-Edge Parameters

Due to the effects of leaf scattering and chlorophyll absorption in the red-edge region (670–750 nm), the spectral reflectance changes dramatically from low to high in this region [38]. In this study, it was found that, in the first three growth stages of winter wheat, the optimal red-edge parameters for the estimation of AGB and LAI were the same for each growth stage, namely REP for the jointing stage, Dr/Dr_min_ for the flagging stage, and REP for the flowering stage. In the filling stage, the optimal red-edge parameters for the estimation of AGB and LAI are REP and Dr/Dr_min_, respectively. The results show that, as for VIs, the optimal red-edge parameters for the estimation of winter-wheat AGB and LAI differ between growth stages. Gao et al. [61] found that red-edge parameters are less effective than the vegetation indices for estimating the LAI. This result is consistent with the findings of this study. The reason for this phenomenon is that, due to the spectral resolution of the sensor, the red-edge region has a strong sensitivity to crop parameters. The calculation of red-edge parameters requires a sensor with a high spectral resolution [62,63]. Additionally, the measurement of these parameters is affected to a certain extent by the pigment content of leaves. It is necessary to further study the connection between RPs and crop parameters.

### 4.3. Estimation of AGB and LAI Based on Vegetation Indices and Red-Edge Parameters

In this study, it was found that, although estimations of AGB and LAI based on red-edge parameters alone were inferior to those based on VIs alone, estimations of AGB and LAI based on a combination of VIs and red-edge parameters were superior to those based on only VIs or only red-edge parameters (Table 4, Table 5 and Table 6). Furthermore, using this combined approach, higher estimation accuracy was obtained for AGB than for LAI. AGB reflects the canopy coverage of the crop. As the crop grows, AGB increases from the jointing to the filling stage; meanwhile, LAI is related to AGB and water content [23,64]. From the jointing stage to the filling stage, LAI first increases and then decreases. 

### 4.4. AGB and LAI Estimation Performance of Regression Methods 

As shown in Table 7 and Table 8, the estimates of AGB and LAI obtained using a combination of VIs and red-edge parameters and using either the SWR and PLSR methods are more accurate than the estimates obtained using the optimal vegetation indices or the optimal red-edge parameters. Of the two regression methods, overall, the PLSR method obtained superior estimates of the two crop parameters. Additionally, it was found that the validation results were acceptable and consistent with the performance of the modeling (Figure 3, Figure 4, Figure 5 and Figure 6). The superior performance of the PLSR method observed in this study is consistent with previous results. For example, Yue et al. [65] used the PLSR regression technique to estimate the AGB of winter wheat, and obtained accurate estimates, while Fan et al. [66] improved the accuracy of nitrogen content estimation using PLSR. As the PLSR method uses principal component analysis, and can solve the problem of collinearity among multiple variables. Therefore, a model based on VIs, red-edge parameters, and the PLSR method was used in order to produce estimated distribution maps of AGB and LAI for the 48 study plots. The estimated values are consistent with the ground-measured values, indicating that the model is reliable.

## 5. Conclusions

In this work, UAV-based hyperspectral remote sensing images were used to estimate the AGB and LAI of winter wheat at different growth stages based on vegetation indices, red-edge parameters, and their combination. It was found that the estimates obtained using a combination of vegetation indices and red-edge parameters were superior to those obtained using a single vegetation index or red-edge parameter. Furthermore, it was found that the estimates obtained using a combination of vegetation indices and red-edge parameters and either the SWR or PLSR methods were more accurate than those obtained using linear regression and only vegetation indices or red-edge parameters, and that the PLSR method obtained higher accuracy and stability than the SWR method.

## Figures and Tables

**Table 1:** Main attributes of the Ultra high definition (UHD) 185-Firefly hyperspectral sensor.

Country of Origin	Germany
Dimensions	195 mm × 67 mm × 60 mm
Weight	470 kg
Spectral range	450–950 nm
Number of channels	125
Spectral sampling interval	4 nm

**Table 2:** Vegetation indices used in this study.

Vegetation Indices	Definition	References
PBI (plant biochemical index)	R810/R560	[39]
LCI (linear combination index)	(R850 − R710)/(R850 + R670)^1/2^	[40]
PSSR (pigment-specific simple ratio)	R800/R500	[41]
RARS (ratio analysis of reflectance spectra)	R760/R500	[42]
WDRVI (modified wide dynamic range vegetation index)	(0.1 × R800 − R670)/(0.1 × R800 + R670)	[43]
PSND (pigment-specific normalized difference)	(R800 − R470)/(R800 + R470)	[41]
MSR (modified simple ratio index)	(R800/R760 − 1)/(R800/R670 + 1)^1/2^	[44]
NDVI (normalized difference vegetation index)	(R800 − R680)/(R800 + R680)	[45]
SR (simple ratio vegetation index)	R750/R550	[46]
PSRI (plant senescence reflectance index)	(R680 − R500)/R750	[47]
NPCI (normalized pigment chlorophyll ratio index)	(R670 − R460)/(R670 + R460)	[48]
GI (greenness index)	R554/R677	[49]
OSAVI (optimized soil adjusted vegetation index)	1.16×(R800 − R670)/(R800 + R670 + 0.16)	[50]
RDVI (renormalized difference vegetation index)	(R800 − R670)/(R800 + R670)^1/2^	[51]
RVSI (red-edge vegetation stress index)	[(R712 + R752)/2] − R732	[52]
EVI2 (two-band enhanced vegetation index)	2.5 × (R800 − R670)/(R800 + 2.4 × R670 + 1)	[53]
TCARI (transformed chlorophyll absorption ratio index)	3 × [(R700 − R670) − 0.2 × (R700 − R550)(R700/R670)]	[54]
SPVI (spectral polygon vegetation index)	0.4 × [3.7(R800 − R670) − 1.2 × |R530 − R670|]	[55]
TVI (triangular vegetation index)	0.5 × [120(R750 − R550) − 200 × (R670 − R550)]	[26]
MCARI (modified chlorophyll absorption ratio index)	((R700 − R670) − 0.2 × (R700 − R550))(R700/R670)	[54]

Note: R is spectral reflectance.

**Table 3:** Correlation coefficients between crop growth parameters (the above-ground biomass [AGB] and leaf area index [LAe metrics for the estimates of winter-wheat growth parameters (AGB I]) and vegetation indices and red-edge parameters (RPs) for the four growth stages of winter wheat.

Spectral Parameter		LAI				AGB			
		Jointing	Flagging	Flowering	Filling	Jointing	Flagging	Flowering	Filling
VI	PBI	0.713	0.722	0.828	0.826	0.708	0.761	0.835	0.700
	LCI	0.695	0.736	0.776	0.817	0.672	0.784	0.772	0.703
	PSSR	0.683	0.731	0.805	0.809	0.671	0.748	0.819	0.681
	RARS	0.675	0.725	0.800	0.806	0.662	0.745	0.818	0.677
	WDRVI	0.669	0.717	0.768	0.812	0.640	0.766	0.784	0.679
	PSND	0.668	0.692	0.765	0.781	0.644	0.736	0.764	0.670
	MSR	0.664	0.725	0.781	0.812	0.639	0.764	0.800	0.680
	NDVI	0.654	0.696	0.721	0.776	0.621	0.759	0.725	0.660
	SR	0.650	0.730	0.797	0.809	0.631	0.754	0.820	0.676
	PSRI	−0.632	−0.682	−0.648	−0.741	−0.593	−0.767	−0.641	−0.624
	NPCI	−0.622	−0.708	−0.716	−0.800	−0.578	−0.752	−0.748	−0.666
	GI	0.529	0.674	0.671	0.748	0.484	0.705	0.703	0.608
	OSAVI	0.494	0.683	0.757	0.775	0.441	0.738	0.774	0.677
	RDVI	0.416	0.655	0.764	0.771	0.365	0.703	0.785	0.680
	RVSI	0.364	0.203	0.338	−0.226	0.407	0.138	0.253	−0.306
	EVI2	0.348	0.642	0.769	0.770	0.295	0.686	0.795	0.684
	TCARI	−0.278	−0.367	0.123	0.026	−0.309	−0.367	0.202	0.067
	SPVI	0.248	0.596	0.769	0.745	0.201	0.632	0.798	0.678
	TVI	0.201	0.508	0.710	0.719	0.150	0.546	0.748	0.641
	MCARI	0.064	0.013	0.398	0.421	0.013	0.051	0.485	0.376
RP	REP	0.680	0.445	0.511	0.664	0.696	0.454	0.473	0.593
	Dr	0.273	0.579	0.766	0.766	0.224	0.628	0.795	0.701
	SDr	0.110	0.562	0.751	0.744	0.151	0.602	0.786	0.670
	Dr_min_	−0.493	−0.532	−0.217	−0.404	−0.501	−0.542	−0.133	−0.243
	Dr/Dr_min_	0.645	0.688	0.579	0.809	0.693	0.725	0.536	0.651

Note: REP: red edge position; Dr: red edge amplitude; SDr: red edge area; Dr_min_: minimum red-edge amplitude.

**Table 4:** Performance metrics for the estimates of winter-wheat growth parameters (AGB and LAI) obtained using the optimal vegetation indices for different growth stages.

Growth Stage	Growth Parameter	Optimal VI	Modeling			Validation		
			*R^2^*	RMSE	NRMSE (%)	*R^2^*	RMSE	NRMSE (%)
Jointing	AGB	PBI	0.47	0.05	20.54	0.60	0.04	14.22
	LAI	PBI	0.55	0.62	17.65	0.62	0.57	14.99
Flagging	AGB	LCI	0.61	0.10	18.86	0.67	0.08	14.76
	LAI	LCI	0.54	1.13	24.75	0.71	0.63	18.41
Flowering	AGB	PBI	0.70	0.11	14.19	0.72	0.09	12.02
	LAI	PBI	0.65	0.72	20.76	0.77	0.48	16.59
Filling	AGB	LCI	0.45	0.21	19.30	0.65	0.15	14.18
	LAI	PBI	0.63	0.56	32.55	0.78	0.34	25.90

**Table 5:** Performance metrics for the estimates of winter-wheat growth parameters (AGB and LAI) obtained using the optimal red-edge parameters for different growth stages.

Growth Stage	Growth Parameter	Optimal RP	Modeling			Validation		
			*R^2^*	RMSE	NRMSE (%)	*R^2^*	RMSE	NRMSE (%)
Jointing	AGB	REP	0.49	0.06	22.57	0.45	0.04	16.87
	LAI	REP	0.57	0.61	17.24	0.41	0.72	18.74
Flagging	AGB	Dr/Dr_min_	0.56	0.10	20.05	0.50	0.09	18.33
	LAI	Dr/Dr_min_	0.51	1.16	25.37	0.54	0.80	23.29
Flowering	AGB	Dr	0.62	0.13	16.04	0.70	0.10	12.46
	LAI	Dr	0.55	0.82	23.46	0.68	0.58	19.81
Filling	AGB	Dr	0.45	0.20	19.29	0.48	0.19	17.32
	LAI	Dr/Dr_min_	0.66	0.53	31.09	0.58	0.48	36.11

**Table 6:** Performance metrics for the estimates of winter-wheat growth parameters (AGB and LAI) obtained using a combination of the optimal vegetation index and the optimal red-edge parameter for different growth stages.

Growth Stage	Growth Parameter	Optimal VI, RP	Modeling			Validation		
			*R^2^*	RMSE	NRMSE (%)	*R^2^*	RMSE	NRMSE (%)
Jointing	AGB	PBI, REP	0.52	0.05	19.46	0.58	0.04	14.78
	LAI	PBI, REP	0.61	0.58	16.42	0.56	0.82	21.34
Flagging	AGB	LCI, Dr/Dr_min_	0.62	0.09	18.65	0.63	0.09	17.02
	LAI	LCI, Dr/Dr_min_	0.55	1.11	24.33	0.66	1.20	34.76
Flowering	AGB	PBI, Dr	0.71	0.10	14.06	0.73	0.11	13.84
	LAI	PBI, Dr	0.65	0.72	20.72	0.77	0.55	18.80
Filling	AGB	LCI, Dr	0.50	0.18	18.42	0.53	0.19	17.91
	LAI	PBI, Dr/Dr_min_	0.69	0.51	29.94	0.72	0.40	29.74

**Table 7:** Performance metrics for the estimation of winter-wheat AGB and LAI obtained using seven vegetation indices (modeling dataset) and either the stepwise regression (SWR) or partial least squares regression (PLSR) method for the four growth stages.

Method	Growth Stage	AGB			LAI		
		*R^2^*	RMSE (kg/m^2^)	NRMSE (%)	*R^2^*	RMSE	NRMSE (%)
SWR	Jointing	0.55	0.05	18.85	0.58	0.60	17.11
	Flagging	0.68	0.09	17.02	0.55	1.11	24.41
	Flowering	0.74	0.11	13.32	0.70	0.66	19.04
	Filling	0.55	0.19	17.53	0.68	0.52	30.21
PLSR	Jointing	0.56	0.05	18.58	0.61	0.58	16.48
	Flagging	0.69	0.09	16.74	0.64	1.00	21.92
	Flowering	0.78	0.10	12.26	0.71	0.65	18.76
	Filling	0.60	0.18	16.53	0.75	0.50	26.81

**Table 8:** Performance metrics for the estimation of winter-wheat AGB and LAI obtained using a combination of vegetation indices and red-edge parameters (modeling dataset) for the four growth stages.

Method	Growth Stage	AGB			LAI		
		*R^2^*	RMSE (kg/m^2^)	NRMSE (%)	*R^2^*	RMSE	NRMSE (%)
SWR	Jointing	0.57	0.05	18.47	0.62	0.57	16.12
	Flagging	0.70	0.08	16.38	0.64	1.00	21.82
	Flowering	0.77	0.10	12.41	0.72	0.65	18.50
	Filling	0.60	0.18	16.51	0.71	0.49	28.63
PLSR	Jointing	0.59	0.05	17.99	0.64	0.56	15.76
	Flagging	0.72	0.08	16.02	0.67	0.96	21.07
	Flowering	0.80	0.09	11.67	0.75	0.61	17.58
	Filling	0.75	0.15	13.11	0.76	0.45	26.17
